# Anthocyanin Encapsulated by Ferulic Acid-Grafted-Maltodextrin (FA-g-MD) Microcapsules Potentially Improved its Free Radical Scavenging Capabilities Against H_2_O_2_-Induced Oxidative Stress

**DOI:** 10.3390/molecules24081596

**Published:** 2019-04-23

**Authors:** Yi Ma, Yunhui Feng, Wanling Zeng, Huibo Luo

**Affiliations:** 1 College of Bioengineering, Sichuan University of Science and Engineering, Zigong 643000, China; zhangyer2008@suse.edu.cn; 2 Department of Physical Education, Guangzhou University, Guangzhou 510006, China; 13795553266@163.com

**Keywords:** antioxidant, oxidative stress, anthocyanin, HT-29

## Abstract

This study aimed to investigate the antioxidant activity and release behavior of anthocyanin (ANC) loaded within FA-g-MD wall (ANC-FA-g-MD microcapsule) in vitro. The microencapsulation of ANC was prepared by spray drying and displayed a biphasic release profile. The combination of ANC and FA-g-MD (0.0625–1 mg/mL) showed a higher antioxidant activity than that of both individuals. A possible intermolecular interaction between ANC and FA-g-MD was studied by UV-vis spectra. Intracellular reactive oxygen species (ROS), 3-(4,5-dimethyl-2-thiazolyl)-2,5-diphenyl-2-H-tetrazolium bromide (MTT) test, and protein expression of quinone oxidoreductase 1(NQO1), glutathione reductase (GSR) and γ-glutamate cysteine ligase catalytic subunit (γ-GCLC) were measured through human colon cancer cells (HT-29). After a 24-hour incubation of the HT-29, the combinations (0–60 μg/mL) exhibited a high potential to diminish the ROS level. And the distinct upregulated expressions of GCLC and NQO1 of HT-29 were detected after treatment with combinations compared to those of single ones. These results suggested that the ANC-FA-g-MD microcapsules exerts enhanced antioxidant effect with capability of the modulation of GCLC and NQO1.

## 1. Introduction

Reactive oxygen species (ROS)-induced oxidative stress has been proven to be implicated in the pathogenesis of many diseases, such as diabetes, cardiovascular disease, and various cancer types [1]. Therefore, it is important to modulate the ROS level using some bioactive compounds with capability of resistance to oxidation. [2,3]. Anthocyanin is a natural pigment, commonly existed in many fruits and vegetables, and presented a good antioxidant activity potential [4,5]. However, the use of anthocyanin as an antioxidant in food is hindered by their vulnerability and poor bioavailability.

So far, many attempts have been made to enhance stability and antioxidant activity of anthocyanins, such as development of delivery systems and multiple antioxidants [6,7]. One approach to enhance bioavailability is microencapsulation. Carbohydrates, protein and their combinations are available for anthocyanin microencapsulation [8,9,10]. These wall materials present physical barriers to protect anthocyanins from degradation. However, they are not capable of resisting to the oxygen radical attack, which may accelerate the degradation of anthocyanins. Just the combination of antioxidants offers an effective alternative to overcome this issue due to their synergistic effect. For example, the multiple antioxidants (β-carotene and anthocyanins) were co-encapsulated into pH-responsive microspheres [6]. The multi-functional microspheres showed a synergistic antioxidant activity accompanying by an enhanced thermal stability. Additionally, numerous studies revealed that the combinations of antioxidants show the better antioxidant effects compared to that of single ones [11,12,13,14]. However, different characteristics of antioxidants may lead to the complex combination operations and low embedding efficiencies. Thus, it is mandatory to develop a simple, effective and nontoxic delivery system to ensure the bioavailability and antioxidant activity of combinations. 

Polysaccharide conjugate, an important encapsulated matrix, provide a good alternative to enhance the antioxidant capacity of ANC [15]. Accordingly, considerable attentions have been paid to develop these functional conjugates. For example, a series of phenolic acids were grafted onto chitosan, starch and cellulose, and their excellent antioxidant properties were correspondingly validated [16,17,18]. In our former study, we developed several new hydroxycinnamoyl maltodextrin derivatives and their antioxidant activity and cytotoxicity were also evaluated [19]. Among them, maltodextrin-grafted ferulic acid (FA-g-MD) showed the best antioxidant activity. Additionally, we found that anthocyanins encapsulated in maltodextrin-grafted-cinnamic acid can significantly enhance the stability of anthocyanins [19]. Bearing these in mind, FA-g-MD can be used as a potential wall matrix to improve stability of anthocyanins. Nevertheless, further studies are necessary to evaluate release behaviors and antioxidant mechanism of anthocyanins microcapsules. To the best of our knowledge, few studies have been reported in regard to biological activity of a delivery system in cell lines. Recently, Amin et al. constructed anthocyanin loaded (PLGA@PEG) nanoparticle system [7], which largely enhanced the in vitro stability and bioactivity of anthocyanin, that is because PLGA@PEG significantly increased the expression level of several redox-regulated enzymes as compared to the bare anthocyanin-treated group. However, no investigation was performed for the antioxidant effect of a combination of modified polysaccharides and anthocyanin in vitro.

Herein, we aimed to evaluate the enhanced antioxidant activity and release profile of ANC (ANC used, delphindin-3-glucoside) loaded with FA-g-MD in vitro. Thus, we studied release profile of the microparticles and investigated the chemical-scavenging or inhibitory effect of FA-g-MD and ANC on 1,1-diphenyl-2-picrylhydrazyl (DPPH) radicals and lipid peroxidation. In addition, we observed the weak interaction among the antioxidants. Furthermore, the effects of the combination of FA-g-MD and ANC on ROS levels were determined. And the modulation of cellular antioxidant defense was studied for the first time by monitoring protein expression of antioxidant enzymes. This study may present advancement towards the formulation of functional foods with enhanced antioxidant activities.

## 2. Results and Discussion

### 2.1. Characterization of Microcapsules

**Figure 1:**
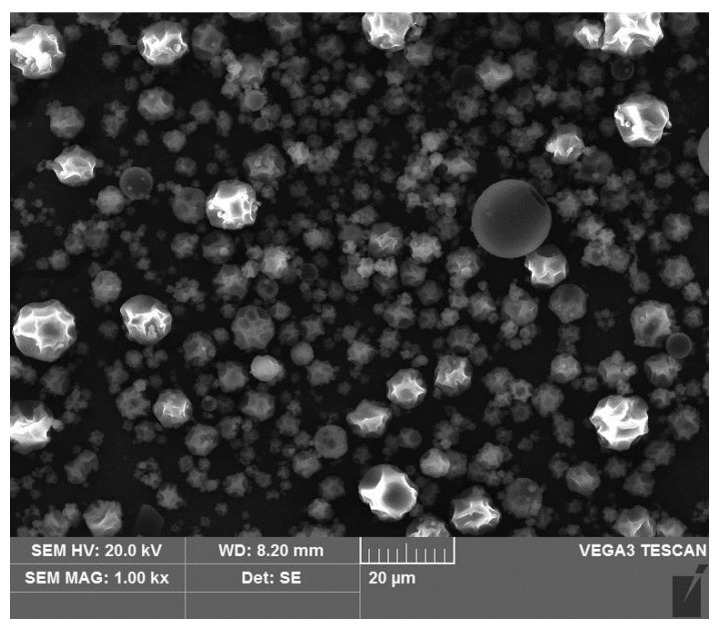
Scanning electron microscope (SEM) images of microcapsules produced by ferulic acid-grafted altodextrin.

ANC loaded with FA-g-MD was produced by spray-dried technique. The morphology of the microcapsules was shown in Figure 1. Most of the microcapsules showed plicated spheres, and some cracks and reliefs were also observed. This morphology could be attributed to the collision of the solid granules during the spray-drying process [20]. The diameter of the microcapsules ranged between 2.07 μm to 14.85 μm with a good dispersity. This observation was previously presented for cinnamic acid-grafted-maltodextrin. The density of the microcapsules were measured to be ranged from 0.571 to 0.587 g/cm^3^. And the encapsulation yield (EY) was 51.3 ± 2.1%, which was due to remain of the prepared FA-g-MD in the spray drier equipment. The value of encapsulation efficiency (EE, calculated using equation in Section 3.4) was 86.3 ± 2.5%. A similar result was reported in a study on the spray-drying anthocyanins using modified MD as wall matrix [21,22]. In addition, water activity and moisture were 0.30 ± 0.01 and 3.47 ± 0.21%, respectively, which are key factors to maintain food quality. These conditions were conducive to the stability of the microcapsules due to less water available in biochemical reactions.

### 2.2. In Vitro Release Profile of ANC

**Figure 2:**
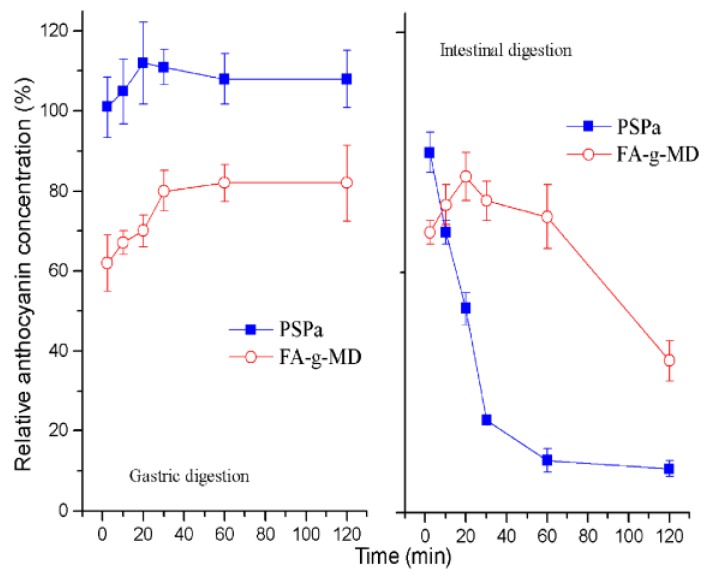
Time-dependent relative anthocyanin concentration in simulated gastric digestion and simulated intestinal digestion after incubation of native ANC and microcapsules produced by FA-g-MD. The values are expressed as mean ± SD (*n* = 3).

To evaluate and compare the release profile of ANC in vitro, the preparations were incubated in simulated gastrointestinal fluid (SGF) and simulated intestinal fluid (SIF). Figure 2 shows the stability of native ANC and the release kinetic from microcapsules. Incubation of these preparations in SGF resulted in an increase of the ANC concentration within the first 20–30 min, and then remained stable for the following 90 min. These results agreed with the release characteristics from the anthocyanin encapsulation systems [23]. It was well known that anthocyanin exists in equilibrium of four molecular species [24]. The high recovery of ANC can be mainly due to the transformation of the colorless chalcone to flavylium cation in gastric environment (pH1.8). The next section of stomach is the small intestine, which was simulated by SIF. During the SIF incubation, the native ANC degraded sharply. The native ANC concentration decreased to 9.2% by the end of incubation time. As expected and previously reported, the major difference between SGF and SIF was attributed to the substantially higher pH of SIF [23]. Compared to the stability of the native ANC, however, the concentration of ANC released from microcapsules increased, indicative of constant release, within the first 20 min. By the end of the incubation time, the ANC concentration declined to 38%. This result indicated that the FA-g-MD microcapsule effectively extended the release of ANC and can be a potential candidate for delivery system of bioactive compounds [7].

### 2.3. Antioxidant Capacity of ANC, FA-g-MD and Their Combination

**Figure 3:**
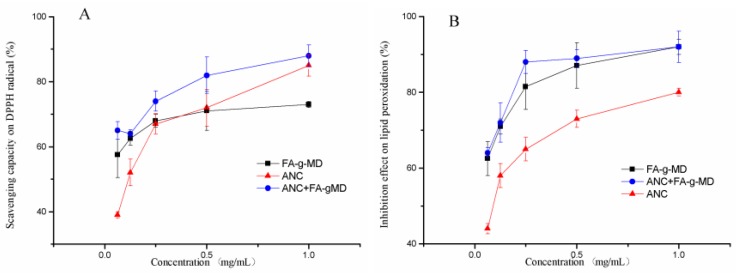
Effects of ANC, FA-g-MD and their combination on inhibiting free radicals. (**A**) DPPH radicals, (**B**) lipid peroxidation. The values are expressed as mean ± SD (*n* = 3).

To evaluate the antioxidant potential, two modes of antioxidant action (hydrogen atom transfer and single electron transfer) can be utilized [25]. As shown in Figure 3A, ANC, FA-g-MD and their combination exhibited a concentration-dependent (0.0625–1 mg/mL) radical-scavenging capacity. Obviously, ANC and FA-g-MD presented good antioxidant activities, as already demonstrated by previous reports [26].These compounds are postulated to have very good electron-donating abilities due to *p*-OH and other electron-donating functional groups. The radical-scavenging capacity of the combination (83 ± 5.6%) was significantly higher than that of ANC (72 ± 5.7%) or FA-g-MD (71 ± 6.1%) (*p* < 0.05).

Furthermore, a dose-dependent lipid peroxidation inhibition effect was observed in Figure 3B. At a concentration of 0.25 mg/mL, the combination showed significantly higher lipid peroxidation inhibition ability (89 ± 2.3%) than that of ANC (73 ± 2.4%) or FA-g-MD (86 ± 6.1%). The similar observations in antioxidant capacity were also detected among the tested samples in the DPPH assay. The difference of antioxidant activity between ANC and FA-g-MD can be attributed to the position of hydroxyl groups and other features in the chemical structure of ANC and FA-g-MD [27]. Generally, ANC loaded with FA-g-MD showed a stronger antioxidant capability compared to that of ANC.

### 2.4. UV-vis Absorption Spectrum of ANC and FA-g-MD

**Figure 4:**
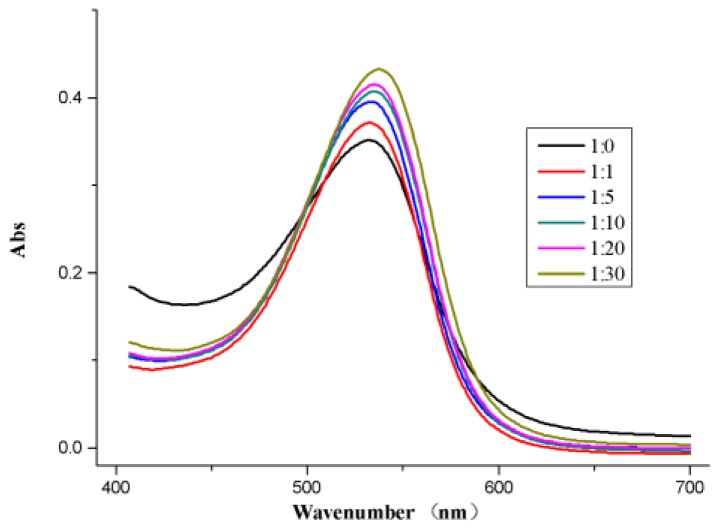
Absorption spectra of ANC (0.5 mg/mL) in the presence of FA-g-MD in a pH 3.5 citrate buffer with dimethyl sulfoxide (DMSO). ANC/FA-g-MD mass ratio: 1:0, 1:5, 1:10, 1:20, 1:30.

In order to investigate interaction between ANC and FA-g-MD, UV-vis absorption of ANC and the combination were measured at various concentrations. As shown in Figure 4, ANC had a maximum absorption peak at 533nm, which is the characteristic absorption of anthocyanin [28]. Moreover, the combination of ANC and FA-g-MD showed a slight bathochromic shift of λ_max_ as the FA-g-MD concentration increased, which clearly indicated some physically intermolecular interactions [29]. This phenomenon resulted from co-pigmentation effect between ANC and FA-g-MD [30], which may attribute to intermolecular H-bonding or π–π stacking. 

### 2.5. Modulation of Cellular ROS Level

**Figure 5:**
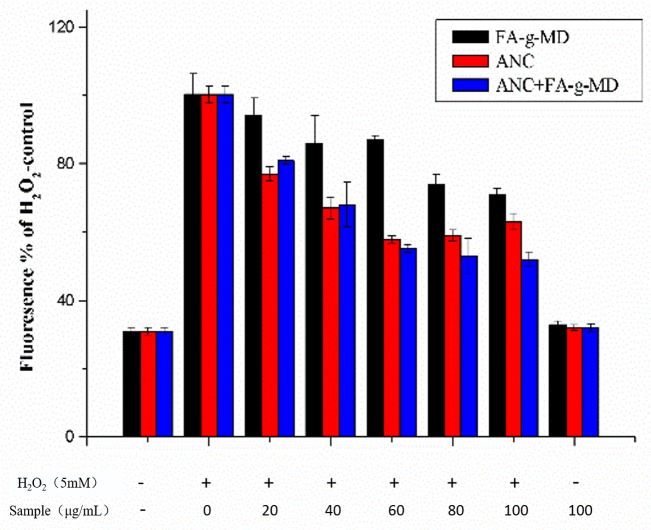
Effects of ANC, FA-g-MD and their combination on reactive oxygen species (ROS) generation. The values are expressed as mean ± SD (*n* = 3).

To analyze of the redox effects of the combination of ANC and FA-g-MD in a cellular system, the cellular antioxidant activities (CAA) were first assessed using the dichlorofluorescein (DCF) assay with HT-29 cells. Tested compounds were used at concentrations of 0–100 μg/mL, because the samples with these concentrations have been proven to be no toxic toward HT-29 cells (data not shown). The influence of ANC, FA-g-MD and their combination on H_2_O_2_-induced ROS level is shown in Figure 5. Generally, ROS levels were significantly higher in the H_2_O_2_-treated groups than that in the control group, whereas the HT-29 cells in the test compound pre-incubated groups showed lower ROS levels than the positive control (samples treated with H_2_O_2_ only), and the ROS levels in HT-29 group only treated with the samples showed no statistical significances compared with the control group. Obviously, FA-g-MD reduced the ROS levels for all samples. At a concentration of 100 μg/mL, FA-g-MD diminished the ROS level down to 70% of the positive control. A slightly different outcomes were obtained for ANC groups. After 24-h incubation of HT-29 cells, ANC (0–60 μg/mL), diminished ROS level in a concentration-dependent manner. At ANC concentration of 60 μg/mL, the fluorescence intensity reduced by 42% compared to the positive control, which was consistent with previous report [5]. Moreover, the combination exhibited a high potential to diminish the ROS levels and showed significantly higher inhibition potential than FA-g-MD at 0–100 μg/mL. In contrast to the effectiveness of this combination, slight inhibition differences were observed between ANC and the combination at the concentration of 20–60 μg/mL. With treatment at 80–100 μg/mL, however, the combination of ANC and FA-g-MD reduced ROS production to a significantly greater extent than ANC did. The highest inhibition by the combination was observed as the reduction down to 48% at 100 μg/mL. Taken together, there was no significant association between chemical (DPPH and lipid peroxidation) and cell-based antioxidant values. In fact, many studies have demonstrated that the differences in the ability of these compounds when incorporated in cells as a requisite for reducing ROS [31,32]. The results suggested that the ANC and FA-g-MD showed ability in the prevention of ROS accumulation. Hence, expression of antioxidant enzymes cannot be ruled out.

### 2.6. Expression of Antioxidant Enzymes

**Figure 6:**
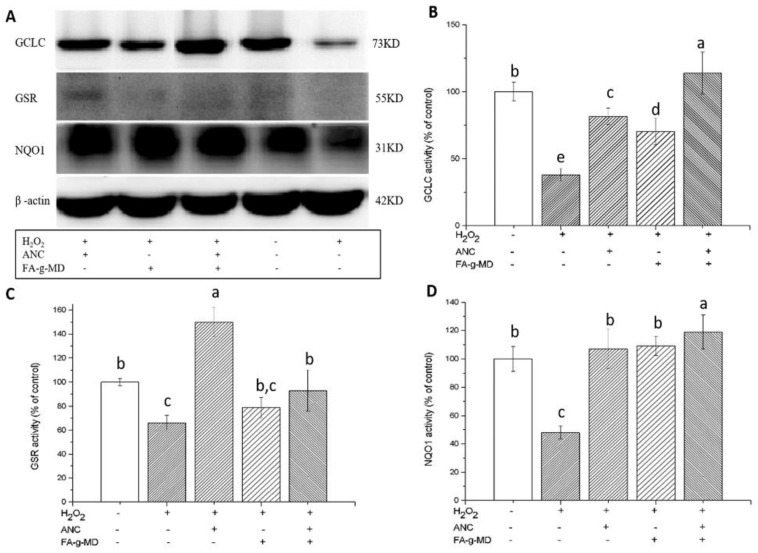
Effects of ANC, FA-g-MD and their combination on the protein expression of GCLC, NQO1, GSR and β-actin in HT-29. (**A**) Western blot representative of three enzyme proteins and control. (**B**–**D**) The values from densitometry of GCLC, GSR and NQO1 (normalized to the level of β-actin). The values are expressed as mean ± SD (*n* = 3). a–e Mean values with unlike letters were significantly difference.

ROS administration is responsible for enzymatic and non-enzymatic antioxidant systems. A well-known antioxidant transcription factor-Nrf2 has been shown to regulate the expression of these antioxidant enzymes, including NQO1, GCLC and GSR [5,33]. To investigate the mechanism of their enhanced antioxidant activity, the modulation of NQO1, GCLC and GSR protein expression in HT-29 cells (24 h-incubation) was studied using Western blotting. As shown in Figure 6, the antioxidant enzyme expression decreased more significantly in the H_2_O_2_ group than in the control group. In contrast, pre-treatment with ANC, FA-g-MD or their combination resulted in the upregulation of NQO1, GCLC and GSR proteins. Therefore, we hypothesized that antioxidant enzymes affect ANC and/or FA-g-MD mediated protection against H_2_O_2_-induced oxidative stress. When the cells were incubated with the combination, GCLC and NQO1 levels showed a stronger increase (*p* < 0.05). For GCLC, there were increase of 140%–180% with the combination compared with that of ANC or FA-g-MDalone. For NOQ1, the incubation with the mixture of ANC and FA-g-MD caused a marginal increase. Taken together, ANC, FA-g-MD and their combination reversed the H_2_O_2_-induced decrease of GCLC and NQO1 and the combination treatment showed evidently higher upregulation than single treatment, which was in accordance with the changes in ROS levels. We supposed that the enhanced antioxidant activity induced by the combination of ANC and FA-g-MD was due to the upregulated expression of GCLC and NQO1. However, although all treatments were significantly better than that of the H_2_O_2_ group, no enhanced effects were observed in GSR expression of the combination treatment. This result may be related to the biphasic response of GSR to ROS levels.

## 3. Materials and Methods

### 3.1. Chemicals, Cells and Media

Delphinidin-3-glucoside (ANC) was purchased from Ziguang (Nanjing, China). FA-g-MD was prepared by our group. 2,2′-diphenyl-1-picrylhydrazyl radical (DPPH) and 2′,7′-dichlorofluorescin diacetate (DCF-DA) were purchased from Aladdin Reagent (Shanghai, China). HT-29 cells were obtained from Chinese Academy of Sciences (Kunming, China). DMEM, DMEM/Nutrient Mix F12 (1:1) medium, fetal bovine serum, penicillin/streptomycin and molecular protein marker were sourced from Invitrogen GmbH (Karlsruhe, Germany). Cell culture consumable materials were purchased from Greiner Bio-One (Essen, Germany). Monoclonal antibody quinone oxidoreductase 1(NQO1), glutathione reductase (GSR), γ-glutamatecysteine ligase catalytic submit (γ-GCLC), and β-actin mouse monoclonal (IgG1) and secondary antibodies (goat anti-mouse IgG1-HRP, goat anti-rabbit IgG-HRP) were purchased from Santa Cruz Biotechnology (Heidelberg, Germany). All organic solvents and other chemicals were of analytical grade and complied with the standards needed for cell culture experiments.

### 3.2. Preparation of Microcapsules

ANC microcapsules were prepared using spray-drying method [10]. Briefly, FA-g-MD was dissolved in hydroethanol. A 2% (*w*/*v*) total solids dispersion with the ratio of FA-g-MD to ANC of 2:1 was acquired. The ANC aqueous solution was slowly added into the wall matrix solutions. Then, the mixtures were homogenized for 10 min at 10,000 rpm using an Ultra T25 Basic (IKA, Staufen, Germany). The dispersions were fed into a Mini Spray Drier B-191 (Büchi, Flawil, Switzerland). The inlet and outlet temperature were 129 °C ± 2 °C and 98 °C ± 2 °C, respectively, with a feed flow of 5 mL/min at room temperature. The prepared microcapsules were kept at 4 °C in a sealed falcon tube to avoid degradation.

### 3.3. Scanning Electron Microscope Analysis (SEM)

Microcapsules morphology analysis was performed on scanning electron microscope (Tescan Co, Brno, Czech) at an accelerating voltage of 20 KV and magnified up to 5000×. The samples were placed on a double-sided adhesive tape. 

### 3.4. Encapsulation Yield (EY) and encapsulation efficiency (EE)

The EY refers to the ratio between the weight of microcapsules and that of all initial solids. And the EE was the ratio between the ANC in the microcapsules (*T_a_*) and the superficial ANC of microcapsules (*S_a_*) [34]. The percentage was calculated as:

$$EE=\left(1-\frac{S_{a}}{T_{a}}\right)\times 100$$

where in the *S_a_* and *T_a_* were determined by absorbance of ANC solution at 523 nm.

### 3.5. Volumetric Density

Volumetric density was measured as previous report [35]. Briefly, 5 g of ample powder were transferred to a 10-mL graduated cylinder and the cylinder was tapped by hand on a bench 100 times from a height of 10 cm. Then, the bulk density was calculated by dividing the mass of the powder by the final volume occupied by the powder in the cylinder.

### 3.6. Moisture Content and Water Activity

The moisture content was measured in Intelligent Water Activity AM-1 (Huayan, China). The moisture was checked by Rapid Moisture Metre (Hengping, China).

### 3.7. In vitro ANC Release

In vitro release kinetics of ANC from microcapsules were evaluated using a published method [36]. Briefly, samples of both native ANC and ANC-loaded capsules were added 100 mL of simulated gastric fluid (SGF: NaCl 9 g/L, pepsin 3 g/L, pH of 1.8) so that the initial total anthocyanin concentration was 0.7%. The solutions were incubated at 37 °C with continuous vibration. 

After a certain time of incubation (0, 10, 20, 30, 60 and 120 min), 1 mL of solutions were withdrawn and centrifuged at 12,000 rpm for 5 min at these time points and followed by the corresponding absorbance of supernatants were respectively recorded. Subsequently, the residual samples were added into 100 mL simulated intestinal fluid (SIF: NaCl 9g/L, pancreatin 10g/L, trysin 10 g/L, bile salts 3 g/L, pH of 6.5) and incubated at 37 °C and continuously agitated. At preset time points (0, 10, 20, 30, 60 and 120 min), the samples were centrifuged at 12,000 rpm for 5 min to separate the dispersed components. The resulting supernatants were measured as spectrophotometric method. 

### 3.8. DPPH Radical Scavenging Ability Assay

DPPH radical scavenging ability of all three samples was evaluated by the method according to the previous report [37]. First, ANC, FA-g-MD and ANC@ FA-g-MD were dissolved into 1.5 mL DPPH solutions to acquire samples solutions with concentration of 1250, 625, 250, 125 and 62.5 μg/mL respectively. DPPH replaced with DMSO for the above-mentioned solutions were taken as controls. All the sample solutions and controls were allowed to stand at room temperature for 30 minutes. Then, the absorbance of solutions samples and controls at 517 nm were recorded. Finally, the scavenging effect was defined by the following equation:

Scavenging effect (%)=[1−(Asample−Acontrol)/Ablank]×100

where *A_sample_*, *A_blank_* and *A_control_* are the absorbance of the sample, blank and control, respectively.

### 3.9. Assay of Lipid Peroxidation Inhibition

The thiobarbituric acid reactive substances assay was used to determine the lipid peroxidation inhibitory effect according to the previous report [38]. Briefly, the lipid-rich medium was obtained by mixing 1.0 g of mouse liver and 100 mL of purified water. And then 1 mL of liver homogenate, 0.2 mL of different samples solutions or controls, 50 μL of FeCl_2_ solution (0.1 mM) were uniformly mixed and incubated at 37 °C for 1 h. Subsequently, 0.3 mL of trichloroacetic acid (20%, *w*/*v*) and thiobarbituric acid (0.8%, *w*/*v*) mixtures were added to terminate the reactions. The resulting solution was incubated at 100 °C for 15 min and then centrifuged (6000 rpm, 5 min). The absorbance of the supernatant was measured at 532 nm. The inhibitory effect on lipid peroxidation was expressed as the following equation:

Inhibitory effect (%)=[1−(Asample−Acontrol)/Ablank]×100

where *A_sample_*, *A_blank_* and *A_control_* are the absorbance of the sample, blank and control, respectively.

### 3.10. UV-vis Analysis

All solutions were prepared in a citrate buffer solution (0.2 M) with DMSO at pH 3.5. 

Various ANC/FA-g-MD solution samples with ANC/FA-g-MD mass ratios (1:0, 1:1, 1:5, 1:10, 1:20 and 1:30) were prepared by adding ANC (0.5 mg/mL) into FA-g-MD solutions. Molecular interaction experiments were performed using a UV-vis spectrometer (Puxi, Beijing, China).

### 3.11. Cell Culture and Viability Assays

HT-29 cells were seeded in high-glucose-containing Dulbecco’s modified Eagle’s medium (DMEM), supplemented with 10% fetal bovine serum and 100 units/mL of penicillin/streptomycin. The cells were cultured at 37 °C in humidified 5% CO_2_ incubator. Cell viability was assessed by MTT assay [39]. Briefly, HT-29 cells were seeded into a 96-well plate at a density of 2 × 10^5^ cells/mL and incubated with ANC, FA-g-MD, or their combination (1, 5, 10, 50 and 100 μg/mL) for 24 h. Subsequently, the chemical medium was removed, and the cells were placed in the fresh MTT solutions for 4 h at 37 °C. The optical density was measured at 570 nm using BMG LAB TECH FLUOstar (GmbH, Offenburg, Germany).

### 3.12. ROS Assay

Oxidative stress in HT-29 cells was assessed using the DCF assay [40]. Briefly, cells were cultured in 96-well plates and cultivated with ANC, FA-g-MD, or their combination for 24 h. After washing with 100 μL of PBS, cells were incubated for 30 min with 50 mM DCF-DA. Then, the treatment medium was removed, and each well was washed with PBS. For the control wells, 100 μL of culture medium without H_2_O_2_ was added into the blank wells. Next, 100 μL of culture medium containing 5 mM of H_2_O_2_ was added into the other wells [41]. The positive control wells contained the cells treated with DCF-DA and H_2_O_2_The fluorescence increase (FI) was measured using a fluorescence microplate reader (Multiscan MK3, Thermo, Beijing, China) at 0 and 30 min (ex/em: 485/528nm). FI was calculated as (F_30min_ − F_0min_) / F_0min_ × 100. 

### 3.13. Protein Expression (Western Blot Analysis)

Western blotting was performed as previous description [42,43]. All the proteins were collected from cells after treatment. 30 μg of protein was electrophoresed on a 10% SDS–polyacrylamide Tris–HCl gel (Bio-Rad, Irvine, CA, USA) and electrophoresed at 170 V for 1 h. The separated proteins were transferred onto a PVDF membrane at 100 V for 30 min on ice. After the transfer, membranes were blocked at room temperature for 1 h in 1 × TBST containing 5% non-fat dry milk. The membrane was probed with primary antibodies (NQO1, γ-GCLC, GSR and β-actin). The signal bands were visualized using enhanced chemiluminescence (Bio-Rad, USA).

### 3.14. Statistical Analysis

Data were expressed as means ± SD. Statistical differences were analyzed using one-way analysis of variance (ANOVA) by IBM SPSS Statistics 20. The differences were considered to be statistically significant when *p* < 0.05.

## 4. Conclusions

To improve the stability and antioxidant activity of ANC, FA-g-MD microcapsules were investigated. These microcapsules effectively extended the release of ANC. Our results have also demonstrated that the combination of ANC and FA-g-MD provided more enhanced antioxidant activity than each alone by scavenging DPPH radical and inhibiting lipid peroxidation directly, diminishing ROS and regulating the antioxidant enzymes. Additionally, the combination function can mainly contribute to the upregulation of GCLC and NQO1. Thus, we assumed that ANC coated with FA-g-MD could be a new antioxidant in the field of manufacturing functional foods to prevent oxidative stress damages.

## Abbreviations

ANC	Anthocyanin
FA-g-MD	Ferulic acid-grafted-maltodextrin
ROS	Reactive oxygen species
DPPH	2,2′-diphenyl-1-picrylhydrazyl radical
DCF-DA	2′,7′-dichlorofluorescin diacetate
NQO1	quinone oxidoreductase 1
GSR	glutathione reductase
γ-GCLC	γ-glutamate cysteine ligase catalytic subunit
